# Inappropriate prescribing in outpatient healthcare: an evaluation of respiratory infection visits among veterans in teaching versus non-teaching primary care clinics

**DOI:** 10.1186/s13756-017-0190-3

**Published:** 2017-03-29

**Authors:** Diane M. Parente, Tristan T. Timbrook, Aisling R. Caffrey, Kerry L. LaPlante

**Affiliations:** 1 Rhode Island Infectious Diseases Research Program, Providence Veterans Affairs Medical Center, 830 Chalkstone Avenue, Providence, RI 02908 USA; 2 Department of Pharmacy Practice, College of Pharmacy, University of Rhode Island, 7 Greenhouse Road, Kingston, RI 02881 USA; 3 Center of Innovation in Long Term Services and Supports, Providence Veterans Affairs Medical Center, 830 Chalkstone Avenue, Providence, RI 02908 USA; 4 Infectious Diseases Division, Warren Alpert Medical School of Brown University, 222 Richmond Street, Providence, RI 02903 USA

**Keywords:** Antibiotic use, Prescribing, Outpatient, Antimicrobial stewardship

## Abstract

A recent study led by the Centers for Disease Control and Prevention (CDC) revealed at least 30% of antibiotic prescriptions in the outpatient setting were inappropriate. In this study of all ages, among adult patients, results were similar to the overall population, with the majority of inappropriate prescribing relating to respiratory infections. We applied the same methodology to investigate rates of antibiotic prescribing for respiratory tract infections in outpatient primary care clinics at the Providence Veterans Affairs Medical Center. The results of our evaluation reflected comparable rates of inappropriate prescribing, but when stratified by teaching versus non-teaching primary care clinics, inappropriate prescribing was significantly higher in non-teaching clinics (17.6% vs 44.0%, *p* < .0001). Respiratory infection visits in non-teaching outpatient clinics may be a pragmatic target for antimicrobial stewardship programs.

**Dear Editor**

We applaud Fleming-Dutra et al. on their recent analysis, which evaluated the rate of inappropriate outpatient antibiotic prescribing across several age groups and infectious diseases [1]. They found at least 30% of outpatient antibiotics prescribed were inappropriate in the United States. Inappropriate prescribing among adults, excluding children, was slightly higher at 33%, with the majority of inappropriate prescription related to acute diagnoses of respiratory conditions including sinusitis, bronchitis, and pharyngitis.

Misuse of antibiotics has been associated with increased morbidity due to adverse drug reactions, increased healthcare costs, and antibiotic resistant bacteria [2]. The Centers for Disease Control and Prevention and the White House, have declared the issue of antibiotic resistant bacteria a threat to the health of the public. As time and resources for antimicrobial stewardship programs (ASPs) are limited, outpatient ASPs need to evaluate which interventions will ensure appropriate prescribing [3]. Currently, data on implementation and metrics of ASP initiatives in the outpatient setting are limited [4], including Veteran clinics [5]. To characterize possible opportunities for an ASP initiative, we performed an evaluation of inappropriate antibiotic prescribing among patients with respiratory tract infections based on the high rate of inappropriate prescribing with these diagnoses described by Fleming-Dutra et al.

Our Veterans Affairs (VA) Medical Center is an academic institution that provides inpatient and outpatient healthcare services to Veterans. The outpatient primary care department consists of 28 prescribers providing care during approximately 34,000 visits per year. Within the primary care department, there are two teaching clinics led by 18 medical residents under the supervision of seven attendings and six non-teaching clinics which consist of two to three primary care prescribers per clinic. Over the time period of this study there were 21 primary care prescribers overall in the non-teaching clinics. As part of our well-established ASP [6–8], we evaluated outpatient primary care oral antibiotic prescribing by diagnosis from March 2013 to February 2015 in Veterans who were 18 years and older. Similar to Flemming-Durta et al., we identified primary care office visits with infection diagnoses codes (ICD-9) of interest from electronic medical records. Antibiotic prescription issue dates were matched to infection diagnosis visit dates and the proportion of oral antibiotics prescribed for each infection diagnosis visit was then calculated. We calculated our rates of prescribing (Table 1), and inappropriate prescribing (Table 2) using methodology described by Flemming-Durta et al. [1] We then compared our data with that from the Flemming-Durta et al. study.

**Table 1:** Rates of prescribing during respiratory infection visits

	Fleming-Dutra et al.^a^	VA clinics total	VA teaching clinics	VA non-teaching clinics
Sinusitis	1206/1748 (69.0)	294/357 (82.4)	38/63 (60.3)	256/294 (87.1)
Pharyngitis	824/1172 (70.3)	123/244 (50.4)	12/53 (22.6)	111/191 (58.1)
Bronchitis	748/1014 (73.8)	431/696 (61.9)	55/168 (32.7)	376/528 (71.2)
Pneumonia	324/478 (67.8)	67/233 (28.8)	15/40 (37.5)	52/193 (26.9)
Total	3102/4412 (70.3)	915/1530(59.8)	120/324 (37.0)	795/1206 (65.9)

All data as no. (%)

*VA* veterans affairs

^a^Unweighted sample data among age ≥20

**Table 2:** Inappropriate prescribing^a^ during respiratory infection visits

	Fleming-Dutra et al.^b^	VA clinics total	VA teaching clinics	VA non-teaching clinics
Sinusitis	150/1748 (8.6)	78/357 (21.8)	0/63 (0)	78/294 (69.1)
Pharyngitis	613/1172 (52.3)	79/244 (32.4)	2/53 (3.8)	77/191 (40.3)
Bronchitis	748/1014 (73.8)	431/696 (61.9)	55/168 (32.7)	376/528 (71.2)
Pneumonia	0/478 (0)	0/233 (0)	0/40 (0)	0/193 (0)
Total	1511/4412 (34.2)	588/1530(38.4)	57/324 (17.6)	531/1206 (44.0)

All data as no. (%)

*VA* veterans affairs

^a^Fleming-Dutra et al. inappropriate prescribing definitions

^b^Unweighted sample data among age ≥20

Prescribing was lower among VA clinic visits (59.8%) compared to non-federal office-based physician visits (70.3%, *p* < .0001). However, when stratified by VA teaching versus VA non-teaching clinics, teaching clinics had markedly less prescribing (37.0% vs 65.9%, *p* < .0001), and non-teaching clinics had a rate similar to that of non-federal office visits (70.3%).

Rates of inappropriate prescribing among patients with respiratory infections were similar among non-federal office-based physician visits and VA clinic visits at 34.2% and 38.4%, respectively. However, VA teaching clinics had significantly lower rates of inappropriate prescribing compared with VA non-teaching clinics (17.6% vs 44.0%, *p* < .0001) and non-federal office visits (17.6% vs 34.2%, *p* < .0001). Our study had several limitations. As with any observational study, misclassification bias may have occurred due to diagnosis coding errors. Since we did not manually review patient electronic medical records and our reports provided limited information, we were not able to separate initial encounters from follow-up visits. It is not uncommon for a patient to be diagnosed and prescribed an antibiotic in the emergency room or hospitalized and upon discharge, follow up with their primary care provider. In addition, we were not able to observe whether patient’s had other co-morbidities that may have influenced the patient’s probability of getting an antibiotic or compare the patients among the different clinic types.

Nonetheless, our data offer insights into inappropriate outpatient prescribing among government clinics as those data were not available from the data source used in the Fleming-Dutra et al. study. While similar rates of inappropriate prescribing for acute respiratory tract infections have been described in two other VA health systems’ outpatient clinics [9], our findings are unique in suggesting that outpatient ASPs may benefit from focusing their efforts in non-teaching clinics, at least among VA clinics. In conclusion, while VA outpatient rates of inappropriate prescribing in respiratory infections were similar to those reported at non-federal office visits, inappropriate prescribing rates among VA teaching clinics were much lower than non-federal office visits. Non-teaching clinics therefore represent an important area of opportunity for ASPs.

## Abbreviations

ASPs: Antimicrobial stewardship programs
VA: Veterans Affairs
